# The power of retrotransposons in high-throughput genotyping and sequencing

**DOI:** 10.3389/fpls.2023.1174339

**Published:** 2023-04-25

**Authors:** Yunus Emre Arvas, Sevgi Marakli, Yılmaz Kaya, Ruslan Kalendar

**Affiliations:** ^1^ Department of Biology, Faculty of Sciences, Karadeniz Technical University, Trabzon, Türkiye; ^2^ Department of Molecular Biology and Genetics, Faculty of Arts and Sciences, Yildiz Technical University, Istanbul, Türkiye; ^3^ Agricultural Biotechnology Department, Faculty of Agriculture, Ondokuz Mayıs University, Samsun, Türkiye; ^4^ Department of Biology, Faculty of Science, Kyrgyz-Turkish Manas University, Bishkek, Kyrgyzstan; ^5^ Center for Life Sciences, National Laboratory Astana, Nazarbayev University, Astana, Kazakhstan; ^6^ Institute of Biotechnology, Helsinki Institute of Life Science (HiLIFE), University of Helsinki, Helsinki, Finland

**Keywords:** molecular markers, interspersed repeats, amplification profiling, inter-retrotransposon amplified polymorphism, transposable elements, retrotransposon

## Abstract

The use of molecular markers has become an essential part of molecular genetics through their application in numerous fields, which includes identification of genes associated with targeted traits, operation of backcrossing programs, modern plant breeding, genetic characterization, and marker-assisted selection. Transposable elements are a core component of all eukaryotic genomes, making them suitable as molecular markers. Most of the large plant genomes consist primarily of transposable elements; variations in their abundance contribute to most of the variation in genome size. Retrotransposons are widely present throughout plant genomes, and replicative transposition enables them to insert into the genome without removing the original elements. Various applications of molecular markers have been developed that exploit the fact that these genetic elements are present everywhere and their ability to stably integrate into dispersed chromosomal localities that are polymorphic within a species. The ongoing development of molecular marker technologies is directly related to the deployment of high-throughput genotype sequencing platforms, and this research is of considerable significance. In this review, the practical application to molecular markers, which is a use of technology of interspersed repeats in the plant genome were examined using genomic sources from the past to the present. Prospects and possibilities are also presented.

## Introduction

**Figure 1:**
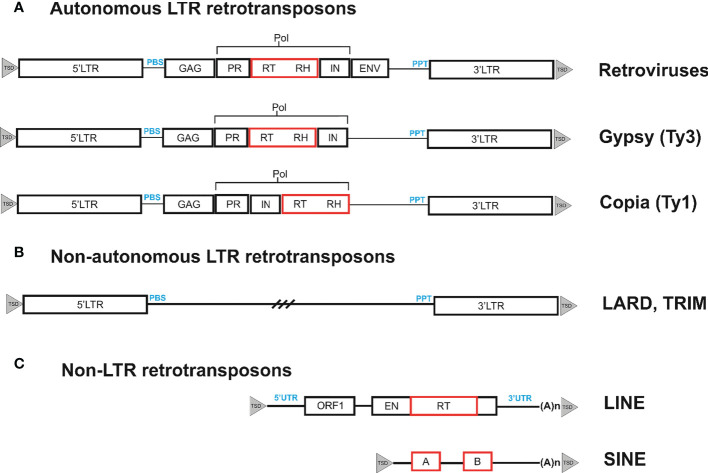
Organization of different types of retrotransposons. **(A)**. Retrotransposons can be classified based on their structural features. One such type is bounded by long terminal repeats (LTRs), which contain the transcriptional promoter and terminator. These LTRs contain short inverted repeats at either end, shown as filled triangles. During reverse transcription, the primer binding site (PBS) and polypurine tract (PPT) domains prime the synthesis of the complementary DNA (cDNA) strand on the (−) and (+) strands, respectively. The internal region of the retrotransposon codes for the proteins necessary for the retrotransposon life cycle. These include the capsid protein (GAG), the aspartic proteinase (AP) that cleaves the polyprotein (AP), the integrase (IN) that inserts the cDNA copy into the genome, and the reverse transcriptase (RT) and RNaseH (RH) that together copy the transcript into cDNA. The internal region also contains evolutionarily conserved domains (noted below the element as black boxes) that are necessary for function and can be used to isolate retrotransposons from previously unstudied plant species. These conserved domains can be targeted for amplification and sequencing of retrotransposons from a variety of species. The LTRs are generally well-conserved within families and can serve as targets for primer design to generate DNA footprints. DNA footprints are useful for studying the evolutionary history and diversity of retrotransposons within and between species. **(B)**. Non-autonomous LTR retrotransposons have two types of non-autonomous forms: Terminal-Repeat Retrotransposons in Miniature (TRIMs) and Large Retrotransposon Derivatives (LARDs). **(C)** Non-LTR retrotransposons, such as LINEs and SINEs, are terminated by a 3′ poly(A) stretch. LINEs have two open reading frames (ORFs) that encode a nucleocapsid protein (gag), an endonuclease (EN), and a reverse transcriptase (RT). The ORFs are flanked by untranslated regions (UTR), with the broken line indicating the 5′ truncations found in many LINEs. SINEs are composed of a tRNA-derived region, an unrelated DNA sequence. The two black boxes labeled A and B depict regions with homology to RNA polymerase III promoters.

The genomes of eukaryotic organisms mostly consist of interspersed repetitive sequences, in particular transposable elements (TEs). In most species studied, interspersed repeats are rather unevenly distributed, with some of them clustered around telomeres or centromeres (Kalendar et al., 2021a). Although TEs exhibit considerable sequence diversity, they can be divided into two well-defined classes according to their structure and propagation strategies. Retrotransposons are the most common type of TEs that belong to class I. Retrotransposons are retrovirus-related genetic elements that amplify through the process of reverse transcription use an RNA-mediated process to transpose, in contrast to class II transposons, which do not require an RNA intermediate (Papolu et al., 2022). Depending on their structure and transposition cycle, retrotransposons can be divided into two main subclasses, long terminal repeat (LTR) retrotransposons and non-LTR retrotransposons, which are based on the presence or absence of LTR at their ends (Wicker et al., 2007). Non-LTR retrotransposons can be further subdivided into long interspersed nucleotide elements (LINE) and short interspersed elements (SINE). Retrotransposons and retroviruses share similarities such as common structural features and basic life cycle stages (**Figure 1**). Retrovirus-like or LTR retrotransposons include endogenous pararetroviruses (Valli et al., 2023), which are remnants of previous rounds of germline virus infection that have lost their ability to reinfect and are fixed in the genome. Each group of transposons has corresponding non-autonomous forms, which are missing one or more genes necessary for transposition. In the case of class II transposons, these non-autonomous forms are known as miniature inverted-repeat transposable elements (MITE), while SINEs are non-autonomous forms of non-LTR retrotransposons. LTR retrotransposons have the following two types of non-autonomous forms: terminal-repeat retrotransposons in miniature (TRIM) and large retrotransposon derivatives (LARD) (Kalendar et al., 2004; Kalendar et al., 2008; Kalendar et al., 2020).

The number of retrotransposons in an organism’s genome is directly proportional to the genome size (Hawkins et al., 2006; Holligan et al., 2006; Jaillon et al., 2007; Ming et al., 2008; Wang and Liu, 2008; Huang et al., 2009; Qiu et al., 2009; Wicker et al., 2009; Kovach et al., 2010; Schmutz et al., 2010; Argout et al., 2011; Shulaev et al., 2011). LTR retrotransposons are the most abundant subclass of retrotransposons in eukaryotic genomes (Bennetzen and Wang, 2014). Retrotransposons are believed to have a vital role in regulating chromosomal structure and structural genes due to their repetitive structure and presence of regulatory signals (Huang et al., 2012; Zervudacki et al., 2018). Eighty percent of the plant genomes of grasses (wheat, barley, and rye) consist of TEs and other repeats (Wicker et al., 2018). The corresponding value for the human genome is 45%; for *Arabidopsis* (Zhang and Wessler, 2004), retrotransposons form 14% of the genome (Turnbull et al., 2018; Akakpo et al., 2020).

Numerous studies have investigated the activities of LTR retrotransposons under stress conditions (Grandbastien, 1998; Pecinka et al., 2010; Tittel-Elmer et al., 2010; Ito et al., 2011; Butelli et al., 2012; Gaubert et al., 2017; Benoit et al., 2019; Cho et al., 2019; Papolu et al., 2021; Ramakrishnan et al., 2022). Mobile elements and endogenous retroviruses are important in the distribution of cis-regulatory elements, which contribute to genetic control in both the short (accumulation of hidden variability and processes of selection) and the long (adaptation and separation) term.

Transposons can insert into different positions within a genome; this leads to changes in the DNA sequence and consequently mutations (Mcclintock, 1950). Retrotransposons can also alter the amount of DNA in the genome by increasing the number of copies of the TE. Transposons have insertional, transcriptional, and translational properties, implying that transposon movements may change depending on the organism, environment, and even tissue specific. However, the movement mechanisms are not completely understood (Rebollo et al., 2011; Schrader and Schmitz, 2019).

TEs have long been considered as agents that give rise to mutations that disrupt gene function after inserting into coding or promoter sequences. These elements play a role in evolution but can also have negative effects on the host organism by disrupting normal gene function (Kalendar et al., 2020). This was demonstrated by Barbara McClintock, who showed that TEs can cause pigmentation loss in corn kernels (Mcclintock, 1950). There has been some opposition to this view; transposition may be beneficial to an organism by mediating epigenetic factors or by acting as cis-acting regulatory regions that exhibit alternative promoters that regulate gene expression (Mirouze and Vitte, 2014; Hirsch and Springer, 2017). Furthermore, amplification of TEs can drive significant biological novelty [i.e. placental pregnancy (Lynch et al., 2011) and innate immunity (Chuong et al., 2016)]; hence, transposition may drive eukaryotic evolution by reshaping gene networks that result in novel features. In crops, TEs have a role in variation of agronomic features, including tomato shape (Xiao et al., 2008) and red pigmentation of apples (Zhang et al., 2019). However, the degree that genetic variation caused by TE can be used for agronomic applications has not been fully verified for all crops (Ramakrishnan et al., 2023).

## Marker-assisted selection (MAS) and historical developments

Molecular genetic markers are short DNA sequences that can be used to identify specific regions of the genome. They can be used in a variety of applications, including genome mapping, disease diagnosis, and classification of individuals or populations. The genetic marker might be either a gene or a sequence that possesses no known functions. Currently, genetic polymorphisms in DNA sequences are analyzed by several strategies, such as various PCR-based methods that detect polymorphisms and PCR-based genome profiling applications: various platforms for hybridization (NanoString Technologies, multiplex ligation-dependent probe amplification [MLPA], microarrays), and next-generation sequencing (NGS). Current analysis strategies have been developed depending on the main methods (**Table 1**).

**Table 1:** Main strategies for detection of molecular genetic polymorphisms.

Marker system	Polymorphism detection strategy	Principle
PCR-based methods detection polymorphism			
Short Tandem Repeat (STR) analysis: variable number tandem repeat (VNTR)	Single-loci polymorphic DNA markers: Simple Sequence Repeats (SSR)	A STR is a microsatellite with repeating units between 2 and 5 bp in length; the number of repeats varies between individuals. This method detects differences in STRs based on PCR product length. Microsatellites and VNTRs can be highly polymorphic and are essential for utility as genetic markers.
Exon-Primed Intron-Crossing (EPIC), Intron Targeting Polymorphism (ITP)	Multiple-loci polymorphic DNA markers	The method relies on the design of primers selected to anneal to highly conserved regions, for example to exons. For illustrative applications, this has been applied to analyze conserved domains within eukaryotic 18S and 28S ribosomal genes and prokaryotic 16S and 23S ribosomal genes to amplify variable intergenic regions known as internal transcribable spacers (ITS) containing the 5,8S ribosomal gene. Intronic regions selected for the determination of polymorphisms are amplified by primers designed for regions close to the exon.
Nucleotide-Binding Site (NBS) profile	Multiple-loci polymorphic DNA markers	Genomic DNA is digested with restriction enzymes after being attached to adapters. Fingerprints of resistance gene regions are generated with the use of adapter-specific and R-gene-specific primers.
Resistance Gene Analog Polymorphism (RGAP)	Multiple-loci polymorphic DNA markers	Analog fingerprints based on resistance genes are amplified with either degenerate specific primers or primer pairs. The primers are designed by targeting the conserved regions of the R genes.
PCR-based genome profiling applications (multiple-loci polymorphic DNA markers)			
Random amplification of polymorphic DNA (RAPD)		Method is based on the use of a single primer (short or standard length) for universal amplification of prokaryotic or eukaryotic genomes (genome profiling). The primer sequence is not essential, thus virtually any primers can be used, including those used for specific amplification of a particular locus. However, PCR amplification conditions should facilitate the formation of multiple amplicons. Generates anonymous markers.
Inter-simple sequence repeat (ISSR)		RAPD-like specific amplification technique for genome profiling; this is a PCR method that uses a single specific primer complementary to the microsatellite sequence. The complementary sequences of two neighboring microsatellite loci are used as primers for PCR; the variant region between them is amplified.
Inter-Primer Binding Site (iPBS)		RAPD-like specific amplification technique for genome profiling; this is a PCR method based on the actually universal occurrence of complement tRNA as a binding site for the reverse transcriptase site in LTR retrotransposons. Primers are annealed to the PBS region of LTR retrotransposons, which are found head-to-head. Amplified products contain LTR and genetic regions.
Inter-Repeat Amplified Polymorphism (IRAP)		RAPD-like specific amplification technique for genome profiling; this is a PCR method based on using a single repeat-specific primer. Many kinds of repeats are dispersed and clustered in the genome, which makes this PCR possible.
Retrotransposon Microsatellite Amplified Polymorphism (REMAP)		RAPD-like specific amplification technique for DNA fingerprinting; this is a PCR method where one of the two primers matches a microsatellite motif with the second specific primers associated with retrotransposons (or any type of repeat sequence). In REMAP, anchored nucleotides (one or more) are used on the 3′ ends of the simple sequence repeat primer to avoid primer drift within the microsatellite sequence.
Palindromic Sequence-Targeted (PST) PCR; Transposon Display (TD)		PCR-based methods combine sequence-specific primers with a universal primer that can anneal to unknown DNA targets, thus ensuring rapid and efficient PCR. This method is based on targeting universal primers to palindromic sequences occurring randomly in natural DNA sequences. PST-PCR involves two rounds of PCR. The first round utilizes a combination of one sequence-specific primer and one universal primer (PST). The second round involves a combination of single- or two-tailed primers; one anneals on a 5′-tail attached to the sequence-specific primer and the other anneals on another 5′-tail attached to the PST primer. The main benefit of PST-PCR is the convenience of using a single-tailed primer for all types of target sequences.
Amplified Fragment Length Polymorphism (AFLP)		A DNA fingerprinting method that utilizes an amplification technique which selectively amplifies a specific subset of digested DNA fragments, resulting in distinctive fingerprints that can be used to compare and analyze genomes of interest. The AFLP protocol involves several key steps. First, the genomic DNA is digested using restriction enzymes, and then adaptors are ligated to the restricted fragments. Next, a preselective PCR amplification is performed to amplify a subset of the restricted fragments. This is followed by a selective PCR amplification, which amplifies only the fragments that have the adaptors and primers that are specific to the target genome of interest. Finally, the amplified DNA fragments are separated using electrophoresis. Variations of the standard AFLP methodology have been developed to target additional levels of diversity, such as transcriptomic variation and DNA methylation polymorphism.
Retrotransposon-Based Sequence-Specific Amplification (S-SAP) Polymorphism (Transposon Display)		S-SAP is a derivative of the Amplified Fragment Length Polymorphism (AFLP) technique and generates amplified fragments containing a retrotransposon LTR sequence at one end and a host restriction site at the other end. Genomic DNA is completely digested, preferably with two different enzymes (usually *Mse*I and *Pst*I) to generate a target for amplification between the retrotransposon sequence and adaptors that are ligated after digestion, using selective bases in the adapter.
Platforms for hybridization			
NanoString Technologies	nCounter	The nCounter technology employs distinctive optical barcodes that hybridize with each target, allowing for the precise digital counting of individual oligonucleotides without the need for any enzymatic steps. These barcodes consist of six fluorophores, which enable highly multiplexed, single-molecule counting of the targets.
Multiplex Ligation-dependent Probe Amplification (MLPA)		Involves a multiplex PCR assay that can employ as many as 50 probes, with each probe specific to a distinct target DNA sequence. These probes consist of two half-probes, namely a 5′ and 3′ half-probe, which comprise a target-specific sequence and a universal primer sequence. This design permits the simultaneous multiplex PCR amplification of all probes. The assay follows several steps, which include DNA denaturation and probe hybridization, followed by ligation and PCR amplification. The amplification products are separated using electrophoresis.
Molecular Inversion Probes (MIP) Technology		The described method involves single-stranded DNA molecules that possess sequences complementary to two areas flanking the target, spanning several hundred base pairs. Once the MIPs bind to the target and hybridize, gap-filling and ligation occur, producing circular DNA molecules that include the target’s sequence, along with adaptors and barcodes. These circularized DNA molecules are then available for subsequent analyses.
Next-Generation Sequencing (Genotyping-by-Sequencing)			
GoldenGate Assay		The extension and amplification steps of the genomic DNA involve a high degree of loci multiplexing (1536-plex) to minimize time, reagent volumes, and material requirements. For each SNP locus, three oligonucleotides are designed: two are specific to each allele of the SNP site, called Allele-Specific Oligos (ASOs), and a third oligo called the Locus-Specific Oligo (LSO) hybridizes several bases downstream from the SNP site. All three oligonucleotides contain regions of genomic complementarity and universal PCR primer sites, while the LSO also has a unique address sequence that targets a particular bead type. During the primer hybridization process, the assay oligonucleotides hybridize to the genomic DNA sample bound to paramagnetic particles. Following hybridization, several wash steps are performed to reduce noise by removing excess and mis-hybridized oligonucleotides. The extension of the appropriate ASO and ligation of the extended product to the LSO join the genotype present at the SNP site to the address sequence on the LSO. The joined, full-length products serve as a template for PCR using universal PCR primers P1, P2, and P3, where P1 and P2 are Cy3- and Cy5-labeled, respectively. After downstream processing, the single-stranded, dye-labeled DNAs are hybridized to their complement bead type through their unique address sequences. Hybridization of the GoldenGate Assay products onto the Array Matrix or BeadChip allows for separation of the assay products in solution onto a solid surface for each individual SNP genotype readout. After hybridization, the fluorescence signal on the Sentrix Array Matrix or BeadChip is analyzed using the BeadArray Reader, which is in turn analyzed using software for automated genotype clustering and calling. No amplification bias can be introduced into the assay, as hybridization occurs before any amplification steps.
Genotyping-in-Thousands by sequencing (GT‐seq)		GT-seq utilizes next-generation sequencing of multiplex PCR amplicons to produce genotypes from relatively small panels (50-500) of target single nucleotide polymorphisms for thousands of individuals on a single Illumina HiSeq lane.
Diversity Arrays Technology (DArT)		Typical procedures include reducing the complexity of genomic DNA using specific restriction enzymes, selecting different fragments to represent parental genomes, PCR amplification, and inserting the fragments into a vector to be inserted as probes in a microarray. Fluorescent targets from the reference sequence can then hybridize with the probes and run through the imaging system.
digitalMLPA		digitalMLPA is a semi-quantitative technology that is used to detect relative copy number variation and identify specific (SNP/InDels) mutations. With digitalMLPA, up to 1000 target sequences can be identified in a single multiplex PCR-based reaction. digitalMLPA produces PCR amplicons that are quantified using Illumina NGS platforms. Sequencing is utilized to detect the number of reads of each digitalMLPA probe amplicon.

Molecular MAS is a method that improves the efficiency of plant breeding by relying on DNA markers to score for specific traits or characteristics. This method allows for earlier selection and reduces the population size of plants, thus saving time and effort. MAS can be used to screen for traits that are difficult or expensive to score phenotypically, such as disease resistance or fruit quality. The use of DNA markers also allows for detection of heredity patterns at the genomic level and the cloning of genes important for natural resistance to disease. This can result in more “green” and cost-effective solutions for disease control. MAS also has the potential to pyramid multiple desirable genes in a new plant variety. By increasing precision in the selection, unwanted side effects in future plant generations can be reduced.

The selection identified by MAS includes scoring in the absence or presence of a plant phenotype of interest that is based upon the DNA banding pattern of connected markers on an autoradiogram or a gel relying on the market framework. The rationale is that the banding pattern provides information about the parental source of the bands in segregants at a marker locus, which illustrates the absence or presence of a specifical chromosomal fragment harboring the allele of interest. The effectiveness of screening in breeding methods is improved in the following several regards. Segregants can be graded at the seedling phase for features that are expressed late in the progress of the plant; this involves features including photoperiod sensitivity, male sterility, and grain quality. It is likely that screening for features that are exceedingly time-consuming, difficult, or expensive to score and determine, such as resistance to biotypes of insects or diseases or certain types or nematodes, tolerance to root morphology, toxicity, mineral, salt deficiencies, and drought. Selection can be applied to certain features concurrently, which is difficult or is not possible *via* traditional means. Heterozygotes are readily defined and separated from homozygotes without referring to progeny testing, which saves effort and time. MAS is a promising choice to be used in improving many phenotypic features of interest in which the evaluation is usually unreliable or expensive. MAS may also increase the efficacy of selection by permitting earlier selection and decreasing the plant population size at the time of selection. Cultivators can rapidly detect heredity patterns at the genome level by directly analyzing the genetic makeup of empirical plants at the seedling stage. It may be useful for features that cannot be defined before plant maturity, such as fruit qualifications and for the features that are difficult to test (such as disease resistance). The selection of resistant plants is performed by using a DNA marker that is connected to the feature that controls the gene rather than turning the resistance of plants to disease into account. To achieve the most economical, environmentally safe, and effective outcomes in disease control, natural resistance genes should be marked in different plant varieties. This approach offers a “green” solution that eliminates the need for expensive chemicals used in disease control. To enhance precision in selection, a uniform practice for scoring involves determining which fragment of each chromosome belongs to each parent and identifying how many genes come from each parent. By increasing precision, undesired side effects can be reduced in the next generation of plants. Additionally, using MAS can help in pyramiding two or more desirable genes in a new plant variety. This approach can further enhance the efficacy of disease control in a cost-effective and environmentally friendly way (Das et al., 2017).

## Interspersed repeats-based genome profiling to study genetic polymorphisms

Interspersed repeats-based genome profiling is a range of different approaches that utilizes the polymorphic nature of TEs to study genetic variations in different plant populations. This approach involves identifying and analyzing the repetitive elements that are interspersed throughout the genome. As mentioned, TEs can insert into different positions within a genome, leading to changes in the DNA sequence and mutation. The emerging heterogeneity in the location of distinct TEs has been exploited for specific molecular marker methods focused on repetitive elements (Bennetzen and Wang, 2014). If integration occurs in the cell line from which pollen or eggs eventually originate, a new polymorphism is formed. These new integrated copies are useful for distinguishing breeding lines, varieties, or plant populations. Changes in the copy number of these repeats and internal rearrangements on both homologous chromosomes occur after the induction of recombination processes during prophase of meiosis (Belyayev et al., 2010). TEs create genomic variation in plants, which has been revealed by certain studies (Kalendar et al., 2000; Belyayev et al., 2010; Wicker et al., 2018; Kwolek et al., 2022). According to a comparative study on different plant genomes, most sequences associated with TEs come from modern insertions (El Baidouri and Panaud, 2013). This implies that the ancient TEs were removed from the genomes and, consequently, there must have been a force that balanced the expansion of the genome caused by TE. This was formulated previously in the “increase/decrease” model (Vitte and Panaud, 2005), which has recently been considered more explicitly with mathematical models (Dai et al., 2018).

## Applications of inter-retrotransposon amplified polymorphisms

**Figure 2:**
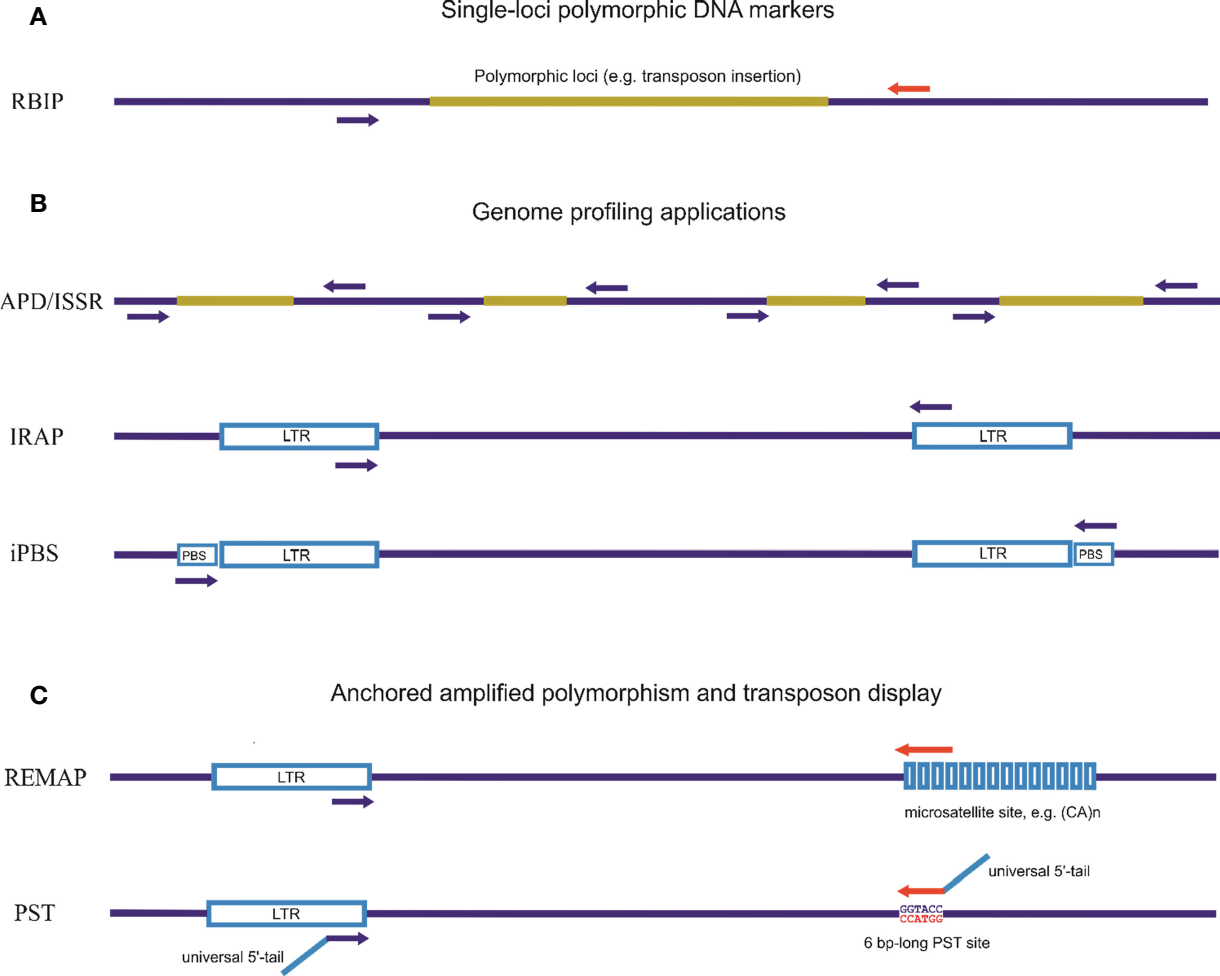
Different approaches for detecting inter-retrotransposon amplified polymorphisms using PCR techniques. **(A)**. Single-loci polymorphic DNA markers. Retrotransposon-Based Insertional Polymorphism (RBIP) is a codominant marker system that uses PCR primers designed for flanking retrotransposon DNA to study insertional polymorphisms in individual retrotransposons. This method detects the presence of mobile element insertions or differences in tandem repeats based on PCR product length. **(B)**. PCR-based genome profiling applications. Random amplification of polymorphic DNA (RAPD) is based on the use of a single primer (short or standard length) for amplification. Inter-simple sequence repeat (ISSR) is a PCR method for DNA fingerprinting using a single primer complementary to the microsatellite sequence. Inter-Repeat Amplified Polymorphism (IRAP) is a PCR method for DNA fingerprinting based on using a single repeat-specific primer. Inter-Primer Binding Site (iPBS) is a PCR method for DNA fingerprinting based on the actually universal occurrence of complement tRNA as a binding site for the reverse transcriptase site in LTR retrotransposons. **(C)**. Anchored genome profiling and transposon display applications using one specific primer for repeat elements in combination with an anchored non-specific primer. Retrotransposon Microsatellite Amplified Polymorphism (REMAP) is a PCR method for DNA fingerprinting where one of the two primers matches a microsatellite motif with second primers associated with retrotransposons (or any type of repeat sequence). Palindromic Sequence-Targeted (PST) and Transposon Display (TD) are PCR-based methods that combine repeat-specific primers with a universal primer able to anneal to palindromic sequences. PST-PCR involves two rounds of PCR. The first round utilizes a combination of one sequence-specific primer and one universal primer (PST). The second round involves a combination of single- or two-tailed primers; one anneals to a 5′-tail attached to the sequence-specific primer and the other anneals to another 5′-tail attached to the PST primer.

Methods of detection of hidden (phenotypically invisible) genetic variations, such as the molecular marker system for genome profiling, were developed based on sequences of multiple families of complex interspersed genomic repeats (**Figure 2**). These genome-profiling PCR techniques for the study of genetic variability in eukaryotes that utilize multicopy and genomic diversity abundance of TEs and endogenous viruses can increase knowledge of genetic relationships and assess the genetic diversity of specific species. Interspersed repeats-based genome profiling applications are a simple PCR method and a cost-effective technique to study individual genetic polymorphisms. Genome profiling is essential as TEs, particularly LTR retrotransposons, are widely distributed throughout the genome and can facilitate recombination events during meiosis (Kent et al., 2017).

The basic principle on which numerous PCR methods for genome profiling have been developed is the use of a single primer specific to the high-copy-number retrotransposon sequences or any other sequences for multiple families of complex interspersed genomic repeats. A second strategy is to use a specific primer to retrotransposon sequences in combination with an anchored primer that may be of varied origin, also including other sequences of interspersed genomic repeats (**Figure 2**).

Eukaryotic genomes harbor many retrotransposon elements, each with their own unique history and level of relatedness. As such, for closely related species, the sequences of a particular retrotransposon will be similar, which reflects the degree of relatedness between species. However, as species become more distantly related, the sequence of a particular retrotransposon will diverge, including the most conserved regions. In the case of closest species, such as within the grass families *Triticum* and *Aegilops*, the sequences of specific mobile elements are almost exactly the same. For species more distant from *Triticum*, such as *Hordeum*, sequences of mobile elements will differ but still retain more than 90% similarity. Therefore, the similarity of mobile elements can be used to study related but distinct species. PCR primers corresponding to the most conserved regions of these mobile elements can also be used (Kalendar et al., 2004; Kalendar et al., 2008; Moisy et al., 2014; Kalendar et al., 2020). One prominent example of such an application is PCR with a single specific primer corresponding to conserved sequences in LTR retrotransposons, which is used for interspersed repeats-based genome profiling. By comparing the sequences of mobile elements across different species, researchers elucidate the evolutionary relationships between those species and the processes that shaped their genomes. In particular, PCR approaches that rely on the identification of transposition element insertion or mutation site polymorphisms include Inter-retrotransposon amplified polymorphism (IRAP) (Kalendar and Schulman, 2006). IRAP is based on PCR amplification of the genomic region between two adjacent LTR retrotransposons oriented in opposite directions. This technique requires a single LTR primer for use in PCR. The PCR products are run on an agarose gel and polymorphisms among the samples are identified based on the banding pattern. IRAP is a technique that is straightforward and rapid. However, the sequences of LTR retrotransposons must be known for this method.

The Inter-Primer Binding Site (iPBS) amplification method is a powerful genomic profiling technique that does not require prior sequence identification of the retrotransposon. The iPBS amplification technique for DNA fingerprinting is a PCR method based on the universal presence of complement tRNA as a binding site for the reverse transcriptase site (PBS) in LTR retrotransposons (Kalendar et al., 2010). Primers are annealed to the PBS region of LTR retrotransposons, which are found head-to-head. Amplified products contain LTR and genetic regions. The LTR primers used in other marker methods are challenging to design, as retrotransposons have no conserved sequences in the LTR regions. On the contrary, many LTR retrotransposons include evolutionarily conserved PBS sequences. The most significant advantage of this method is that it does not need TE sequence information for primer design (Kalendar et al., 2022b). Many studies on TEs are on the determination of relationships using these marker techniques (Monden et al., 2014).

The second strategy for genome profiling and transposon display applications is based on using a specific primer for the repeat element in combination with an anchored non-specific primer. The Retrotransposon Microsatellite Amplified Polymorphism (REMAP) technique for genome profiling is a PCR method in which a specific primer (out of two primers) matches the LTR retrotransposon sequence (or any repeating sequence can be used) and the second anchored primer is annealed to the microsatellite sequence (Kalendar and Schulman, 2006). In REMAP, anchored nucleotides (one or more) are used on the 3′ ends of the microsatellite primer to avoid primer drift within the microsatellite sequence. REMAP and IRAP share similar working principles, as both of require retrotransposon-specific primers whose sequences are known (Hosid et al., 2012).

New methods for high-throughput targeted gene characterization and transposon display have been added to current methodologies, which may be modified to include high-throughput sequencing technologies, among other techniques. For example, Palindromic Sequence-Targeted (PST) PCR utilizes a pair of primers, one of which is complementary to 6-bp long palindromic sequence (PST site) and the other to conserved TE sequences (Kalendar et al., 2019; Kalendar et al., 2021b). The PST-PCR technique allows genome walking and profiling that can be used for the primary characterization of intraspecific and interspecific genetic variability and for screening lines and genotypes.

Retrotransposon-Based Insertional Polymorphism (RBIP) is primarily a codominant marker system that uses one or two pairs of PCR primers designed from combinations of sequences for the retrotransposon and its flanking DNA to study insertional polymorphisms in individual retrotransposons. RBIP is a marker method based on PCR and is used to determine the polymorphisms among two alleles. The comparison of two distinct PCRs permits determination of the polymorphisms. One of the PCRs uses primers that are specific to either retrotransposons or a genomic region near retrotransposons. At the end of the reaction, the interested transposon LTR region is amplified. In the other reaction, two primers specific to the genomic DNA surrounding the retrotransposon are used. The band profiles among the two reactions indicate whether a retrotransposon insertion occurred in the region of interest. RBIP requires sequence information for primer design (Flavell et al., 1998; Kalendar, 2022).

The retrotransposon-based Sequence-Specific Amplification Polymorphism (S-SAP), also known as transposon display, is a modification of the Amplified Fragment Length Polymorphism (AFLP) technique (Vos et al., 1995). This method involves complete digestion of genomic DNA with two different restriction enzymes, typically *Mse*I and *Pst*I, to generate a target for amplification between the retrotransposon sequence and adaptors that are ligated after digestion. The adaptors contain selective bases to facilitate amplification of specific regions. PCR is performed using two primers that are specific to the adapter sequence and the specific mobile element, allowing detection of variations in DNA flanking the mobile element insertion site. The technique uses a multiplex marker system to analyze band profiles among different samples and to detect polymorphisms (Queen et al., 2004). Compared to other retrotransposon marker techniques, S-SAP is costlier and is more difficult to perform. Additionally, the technique requires sequence information for primer generation, which must be designed specifically for the LTR site. However, S-SAP offers greater resolution and accuracy in detecting polymorphisms, making it a valuable tool for genetic analysis.

## Prospects, challenges, and discussion

In plant genetics research and breeding practices, molecular techniques such as genetic characterization, genome profiling, genetic integrity, genetic mapping, feature mapping, MAS, and molecular breeding are widely used. Continuous improvements in molecular marker technology, such as high-throughput genotyping platforms, has led to development of new methods such as the GoldenGate assay, Genotyping-in-Thousands by sequencing (GT-seq) (Campbell et al., 2015), Diversity Arrays Technology (DArT) (Alheit et al., 2011), and NGS-based high-throughput hybridization platform systems (digitalMLPA [Multiplex Ligation-dependent Probe Amplification] and Molecular Inversion Probes [MIP]) (**Table 1**). These advancements have increased efficiency and reduced costs (Elbaidouri et al., 2013; Mir and Varshney, 2012). With such NGS-based high-throughput technological developments, low-throughput molecular markers, such as Kompetitive allele-specific PCR (KASP) (Makhoul et al., 2020), nevertheless remain indispensable for tracking specific genomic regions in molecular breeding programs when analyzing large numbers of samples (Kalendar et al., 2022a; Kalendar et al., 2022c). Therefore, single nucleotide polymorphism (SNP) markers continue to be the most preferred marker systems for development of high-throughput genotypic platforms for genome-wide marker scanning. However, detection of SNP-based markers alone drastically limits the potential to study diversity and genetic polymorphism. One type of polymorphism is the insertion/deletion polymorphism (InDel), which involves the addition or removal of a sequence of different lengths and origins. InDels can have important functional consequences for the chromosome and therefore studying them is crucial. Therefore, other techniques based on mobile element sequences should also be developed, as they can provide complementary information about genomic diversity and evolution.

**Figure 3:**
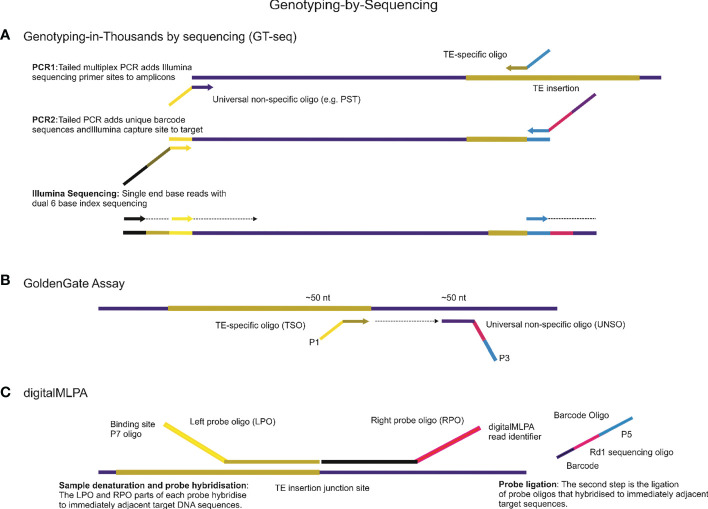
Genotyping-by-Sequencing (GBS) is a powerful technique for detecting genetic variations using Next-Generation Sequencing (NGS) platforms. This technique has several approaches, some of which are highlighted below. **(A)**. One such approach is Genotyping-in-Thousands by sequencing (GT-seq), which uses NGS of multiplex PCR amplicons to produce genotypes from a small panel of mobile element insertions for thousands of individuals on Illumina NGS platforms. The basic principles and steps involved in this technology are also applicable to other NGS applications. **(B)** Another approach is the GoldenGate Assay, which can be designed for genotyping mobile element insertion sites involving a high degree of loci multiplexing (1536-plex). For all mobile element insertions sites, two oligonucleotides are designed, one is TE-specific to the mobile element site, called TE-Specific Oligos (TSO), and the second oligo, called the Universal Non-Specific Oligo (UNSO), for example, a universal primer able to anneal to palindromic sequences (PST), hybridizes downstream from the TE site. All oligonucleotides contain regions of genomic complementarity and universal PCR primer sites, while the UNSO also has a unique address sequence that targets a particular bead type. The extension of the appropriate TSO and ligation of the extended product to the UNSO join the genotype present at the TE site to the address sequence on the UNSO. **(C)** digitalMLPA is a semi-quantitative technology that is used to detect relative copy number variation and identify specific polymorphisms. With digitalMLPA, up to 1000 target sequences can be identified in a single multiplex PCR-based reaction.

Interspersed repeats-based genome profiling is a powerful tool for studying genetic polymorphisms and identifying markers for crop improvement programs. By analyzing the distribution and frequency of TEs within the genome, this method can provide valuable insights into the genetic diversity and evolution of plant populations. In addition to whole-genome sequencing, NGS platforms can also be used for targeted sequencing approaches that focus on specific regions of interest, such as the TEs interspersed throughout the genome. Practically all existing PCR approaches that involve identifying and analyzing repetitive elements can be adapted for use on modern NGS platforms (**Figure 3**). However, when designing primers for NGS-based analysis of repetitive elements, it is important to take into account the specificities of the platform being used. For example, the Illumina HiSeq platform requires the incorporation of adapter sequences in the 5′ tail structure of the primer to facilitate the attachment of the DNA fragments to the sequencing flowcell. One approach that has been successfully adapted for use on NGS platforms is the sequence-specific amplification polymorphism (SSAP) technique, which targets specific retrotransposon insertion sites. The SSAP method involves PCR amplification of the region between two specific primers, one of which is anchored within the retrotransposon and the other in the flanking genomic DNA. The resulting fragments can be sequenced on an NGS platform to identify polymorphisms. Another NGS-based approach for studying TEs is the retrotransposon insertion polymorphisms (RIPs) technique (Du et al., 2022), which uses PCR to amplify specific regions of the genome flanking retrotransposon insertions. The resulting fragments can be sequenced on an NGS platform to identify insertion and deletion events. Overall, the use of NGS platforms for studying TEs offers several advantages over traditional PCR-based methods. NGS allows for the simultaneous analysis of multiple samples and provides greater resolution and sensitivity in detecting polymorphisms. Additionally, NGS platforms can provide information on the structure and organization of TEs within the genome, which can aid in the identification of functional elements and the study of genome evolution.

NGS-based technologies rely on reduced representation sequencing (RRS) techniques (Andrews et al., 2016), including reduced-representation libraries (RRLs), complexity reduction of polymorphic sequences (CRoPS) (Van Orsouw et al., 2007), restriction site-associated DNA (RAD) sequencing (Baird et al., 2008), and low-coverage genotyping. The latter includes multiplexed shotgun genotyping (MSG) and genotyping by sequencing (GBS), which are innovative methods used in genomics (Elshire et al., 2011). These methods can provide informative results even when the reference genome is not available. The choice of method depends on the specific requirements of the study (Mir and Varshney, 2013). As more genome sequences become available, it will be necessary to develop new technologies that allow rapid exploration of the diversity of allelic gene variants in cultivated species that correspond to important plant physiological traits (Springer and Jackson, 2010). In conclusion, NGS-based analysis of repetitive elements is a powerful tool for genome profiling and can provide valuable insights into the genetic diversity and evolution of plant populations. However, careful consideration must be given to the specificities of the sequencing platform and the design of the primers used to ensure accurate and efficient sequencing.

Multiple NGS platforms and “omics” (i.e. genomics, proteomics, transcriptomics, epigenomics, and metabolomics) technologies offer many advantages and can therefore be used as high-throughput genotyping platforms. NGS platforms have revolutionized genomic approaches and have drastically reduced the time and cost required to obtain a DNA sequence. Markers can then be used for various applications, such as population genetics, association studies, and GWAS. The discovery of high-throughput genetic markers and the use of restriction enzymes for genotyping have several advantages and will become the methods of choice for marker research (Kalendar et al., 2022b). One of the advantages of these methods is that they can be used both for model organisms with high-quality reference genome sequences and for non-model species that do not have existing genomic data. Using these evolving technological methods, linkage mapping or (Quantitative Trait Locus) QTL may identify recombination breakpoints and genomic regions that are differentially expressed among populations for quantitative genetic research, genotype progenies for MAS, or resolve phylogeographies of wild populations.

Omics technologies that promise to detect tissue-specific changes with increased sensitivity and allow simultaneous analysis of thousands of genes, proteins, or metabolites (Kroeger, 2006) will increasingly provide sufficient data to create many digital platforms. The integration of new omics technologies with traditional breeding methods is important for seed production in the agriculture industry, as it helps plant growers make informed decisions based on genetic and molecular information. This combination of technologies will assist in meeting commercial criteria for seed production (Flavell, 2010; Li et al., 2018). Traditional plant breeding methods that rely exclusively on phenotypic mapping of desirable traits have limitations in defining gene-trait relationships (Flavell, 2010). The integration of modern omics technologies with traditional breeding can help identify specific gene functions related to seed development, leading to improved seed quality in economically important crops. The use of these cutting-edge technologies can benefit the modern seed industry by providing better tools for seed production (Toubiana and Fait, 2012). Studying the activity of genes at specific plant growth stages, such as grain filling or embryogenesis, may reveal critical components that regulate important metabolic processes that can be used to improve seed quality (Thompson et al., 2009).

## Conclusion

TEs are a core component of all eukaryotic genomes, each with its own unique history and level of relatedness within the same species and between related species. Retrotransposons are widely present throughout the genome, and their replicative transposition allows them to insert themselves into the genome without removing the original elements. For closely related species, the sequences of a particular retrotransposon will be similar, reflecting the degree of relatedness between these species. Various applications of molecular markers have been developed to exploit the fact that these genetic elements are ubiquitous and their ability to be stably integrated into dispersed chromosomal localities that are polymorphic within a species. The ongoing development of molecular marker technologies is directly related to the deployment of high-throughput genotype sequencing platforms, and this research is of considerable significance. Digital NGS-based platforms can be used to study the transposition and site-specific recombination of TEs. Genotyping by sequencing includes a wide range of approaches for detecting genetic variations, and each of these approaches has unique advantages and limitations. Nonetheless, the advances in NGS technologies have greatly improved the ability to investigate the diverse nature of polymorphisms and their role in phenotypic variation. These platforms allow for high-throughput and cost-effective analysis of large amounts of genomic data, which can be used to identify and characterize TEs and their impact on the genome. This information can then be used for various applications, such as genetic characterization, genetic mapping, and marker-assisted selection.

## Author contributions

RK, SM, YA, and YK wrote the manuscript. All authors reviewed the manuscript. All authors contributed to the article and approved the submitted version.
